# A Novel Chemotaxis Assay in 3-D Collagen Gels by Time-Lapse Microscopy

**DOI:** 10.1371/journal.pone.0052251

**Published:** 2012-12-19

**Authors:** Angela Vasaturo, Sergio Caserta, Ilaria Russo, Valentina Preziosi, Carolina Ciacci, Stefano Guido

**Affiliations:** 1 Dipartimento di Ingegneria Chimica Università di Napoli Federico II, Naples, Italy; 2 CEINGE – Advanced Biotechnologies, Naples, Italy; 3 Gastrointestinal Unit Baronissi, School of Medicine, University of Salerno, Salerno, Italy; University of Birmingham, United Kingdom

## Abstract

The directional cell response to chemical gradients, referred to as chemotaxis, plays an important role in physiological and pathological processes including development, immune response and tumor cell invasion. Despite such implications, chemotaxis remains a challenging process to study under physiologically-relevant conditions in-vitro, mainly due to difficulties in generating a well characterized and sustained gradient in substrata mimicking the in-vivo environment while allowing dynamic cell imaging. Here, we describe a novel chemotaxis assay in 3D collagen gels, based on a reusable direct-viewing chamber in which a chemoattractant gradient is generated by diffusion through a porous membrane. The diffusion process has been analysed by monitoring the concentration of FITC-labelled dextran through epifluorescence microscopy and by comparing experimental data with theoretical and numerical predictions based on Fick's law. Cell migration towards chemoattractant gradients has been followed by time-lapse microscopy and quantified by cell tracking based on image analysis techniques. The results are expressed in terms of chemotactic index (I) and average cell velocity. The assay has been tested by comparing the migration of human neutrophils in isotropic conditions and in the presence of an Interleukin-8 (IL-8) gradient. In the absence of IL-8 stimulation, 80% of the cells showed a velocity ranging from 0 to 1 µm/min. However, in the presence of an IL-8 gradient, 60% of the cells showed an increase in velocity reaching values between 2 and 7 µm/min. Furthermore, after IL-8 addition, I increased from 0 to 0.25 and 0.25 to 0.5, respectively, for the two donors examined. These data indicate a pronounced directional migration of neutrophils towards the IL-8 gradient in 3D collagen matrix. The chemotaxis assay described here can be adapted to other cell types and may serve as a physiologically relevant method to study the directed locomotion of cells in a 3D environment in response to different chemoattractants.

## Introduction

The ability of cells to migrate, adhere, and change shape, which is fundamental for all eukaryotes, is primarily regulated by external signals, although there are instances when cells respond to internal cues as well. One of the most interesting and relevant cases of cell migration in response to external stimuli is chemotaxis, i.e. the directional movement of cells along a concentration gradient. Chemotaxis is implicated in a range of physiologically relevant phenomena such as inflammatory response [1], homeostatic circulation, and development [2]. It also concerns a number of disorders and pathological processes including infectious and allergic diseases, wound healing [3], angiogenesis, atherosclerosis, and tumor dynamics [4]–[6]. In the latter case, it is well known that cancer cells can migrate both individually and in a collective manner [7]. Moreover, it has been recently shown that a diffusional instability mechanism [8] can induce the separation of single or clustered cells from the main tumor body, which can then migrate toward the source of nutrients, e.g. a blood vessel, thus invading wider areas and tissues. A still open issue is how soluble gradients might be continuously maintained *in vivo*, where it is known that several physical events such as muscular contraction, convection of extravascular fluid, and lymphatic flow might perturb the graded diffusion of soluble substances.

Despite its ubiquity and importance, chemotaxis remains a difficult process to investigate in a quantitative way, partly because it occurs in complex 3D environments not easily reproducible *in vitro* and not readily compatible with live cell microscopy.

Early efforts to generate spatially linear and temporally stable chemical gradients led to the development of diffusion-based chambers. In these assays, a gradient is established by diffusion inside a porous medium or through a small gap between two large reservoirs containing chemoattractant solutions of different concentrations. Among the first commonly used cell migration assays, the Boyden chamber [9] and the under agarose assay [10] are easy to use, but do not allow cell migration to be monitored as a function of time and do not provide well defined concentration gradients. Microfluidic devices, usually fabricated in PDMS (PolyDiMethylSiloxane) by soft lithography [11]–[15], have also been recently proposed as a tool to observe cell behaviour and migration under chemotaxis or interstitial flow conditions. Convective and diffusive transport can be decoupled by using microfluidic agarose membranes; the effect of shear stress can be also investigated by exposing the cells to static or pulsating flows [16], [17]. Two compartments, containing the chemoattractant and the cells, respectively, are connected side by side horizontally in the Zigmond chamber [18] or as concentric rings in the Dunn chamber [19]. In a recent modification of this technique [20], gradients with defined directions are maintained for at least 24 hours. These assays are typically meant for migration on 2D substrata. Direct observation chambers where the chemoattractant solution is in contact with a 3D gel containing cells have also been reported [21], [22], but quantitative control of the concentration gradient was difficult to achieve. 2D assays are easy to handle and provide important tools for understanding the migratory activity in response to natural or pharmacological modulators, but there could be different mechanisms in 2D vs 3D cell migration [23]–[25], the latter being in principle more adequate to mimic the *in vivo* environment.

An ideal *in vitro* assay of cell chemotaxis should be performed in a tissue-like collagen or fibrin gel, allowing direct cell tracking [26] and determination of the concentration gradient of the chemotactic factor within the gel, and be relatively simple to set up with significant reproducibility. Since cells are able to sense a spatial increase in chemokine concentration to direct their motion, chemotaxis studies require a way to deliver chemicals to cells in a controlled fashion. These criteria have been fulfilled in the *in vitro* assay of leukocyte chemotaxis reported by Moghè et al. [27], in which the cells are initially dispersed throughout the gel rather than concentrated on the filter surface as in the Boyden chamber, thus minimizing cell – cell interactions and cell alteration of the chemotactic factor (CF) gradient. A simple modification of this leukocyte chemotaxis assay reported by Tranquillo et al. [28] allows a gradient of similar steepness to be realized for sufficiently long periods to assay chemotaxis of slow moving cells, such as fibroblasts. It involves the placement of a barrier between the two halves of a chamber (one-half initially containing CF at uniform concentration, the other initially containing none), leaving a small gap at one end of the barrier that serves to geometrically (or dimensionally) constrain the free diffusion; this small gap hinders the passage of the diffusing molecules, thereby slowing the decay rate of the spatial gradient, which emanates radially outward.

In this paper, we present a novel chemotaxis assay in 3-D collagen gels based on a direct-viewing chamber that is autoclavable and reusable, and can be coupled to or integrated with a time-lapse video microscopy and image analysis workstation. In our chamber a chemoattractant concentration gradient in the collagen gel sample seeded with cells is generated by diffusion through a porous membrane. The proposed technique allows the comparison of chemotactic response with control data of random motility (i.e. in the absence of any concentration gradient) of the same cells during the same experiment. Cell migration, either in un-stimulated conditions or under the action of the chemoattractant gradient, is observed by time-lapse microscopy. Cell tracking is performed off-line by image analysis and the results are expressed in terms of a chemotactic index and velocity. The diffusion process was preliminarily monitored by fluorescence microscopy of FITC-labelled dextran and analysed numerically by finite elements. The assay has been tested by using human neutrophils and Interleukin-8 (IL-8), an important neutrophil chemotactic factor [29]–[31] and member of the family of chemokines, which are small basic endogenous peptides (8–14 kDa) [32] responsible for the directed migration of leukocytes from the bloodstream into surrounding tissues [29], [33], [34]. Overall, our methodology provides a combination of features not currently available in other assays, such as live cell imaging with both low and high resolution optics, a 3D matrix, quantitative data analysis based on cell tracking, a well characterized concentration gradient lasting for extended time periods, an autoclavable chamber simple to operate with an integrated control well, and the possibility of testing different cell lines and chemoattractants.

## Materials and Methods

### Ethics Statement

The work was approved by the ethics committee of the University of Naples Federico II (PT1058/12). The research was entirely conducted in Italy. Two authors of this manuscript served as human donors of samples used in the experiments and were designated as A and B. Human donors gave written informed consent to participate to the study.

### Neutrophil isolation

Peripheral blood (10 ml) was taken from healthy human donors into BD Vacutainers containing K3EDTA. Neutrophils were freshly isolated by dextran sedimentation and centrifugation on Ficoll-Hypaque [35]. The pellet of a density-gradient centrifugation containing neutrophil granulocytes and erythrocytes was diluted 1∶1.3 with a high-molecular-weight dextran solution. After 2 h erythrocytes had settled down and the neutrophil granulocytes containing supernatant was separated from the pellet. When necessary, red blood cells in the neutrophil-rich fraction were lysed with hypotonic saline. The neutrophils were washed twice with phosphate buffered saline (PBS) and then resuspended in RPMI 1640 containing 10% heat inactivated fetal bovin serum (FBS). The obtained purified neutrophil granulocytes were used immediately after isolation. The resulting cells contained approximately 96.4% neutrophils, as assessed by microscopy with Wright-Giemsa staining [36].

### Chemotaxis Chamber

**Figure 1:**
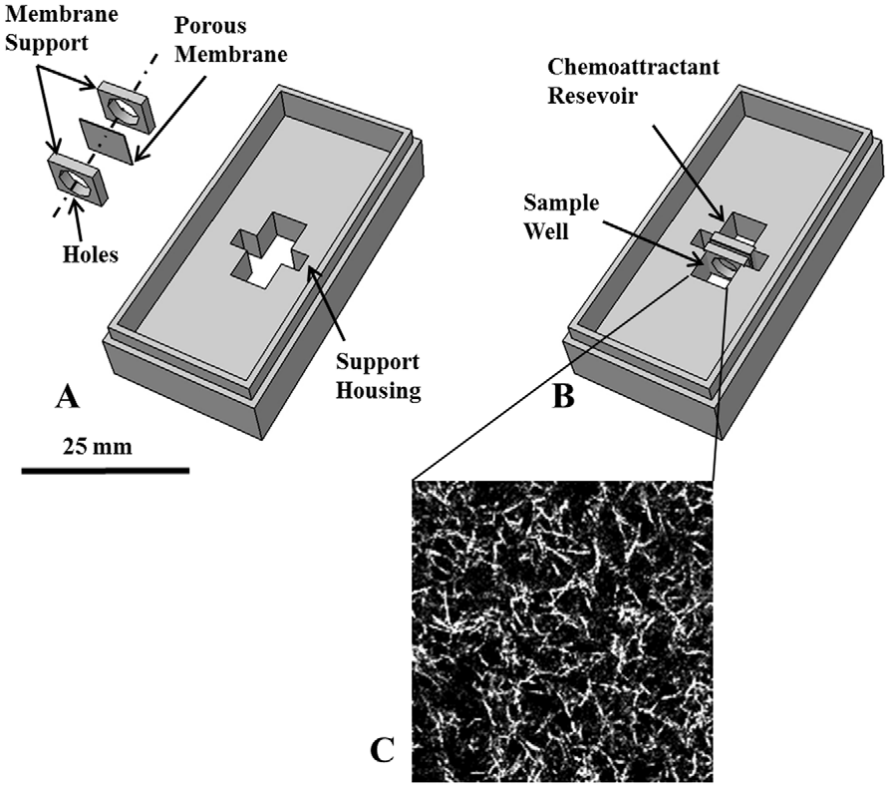
Chemotaxis chamber. A: In the exploded view rendering of the chamber all the components are individually visible. B: In the assembled rendering, the membrane, sandwiched between two aluminum frames, is housed in the chamber, separating the sample well and the chemoattractant reservoir. C: Typical collagen gel morphology in confocal microscopy (63×, oil). Image size is 50 microns.

The chamber was designed to maintain both cell viability and good optical quality over a time scale of 24 h, and to be easy to use and handle. The chamber consists of a single aluminium block glued on top of a microscope slide by using a silicone adhesive resistant to high temperatures. The chamber can then be autoclaved for sterilization and reused. A porous membrane (0.22 µm pores, Millipore), sandwiched between two rectangular metal frames, separates two compartments, one for the cell seeded collagen gel (sample well), and the other as a reservoir of the chemoattractant solution (chemoattractant well). During the experiment the chemoattractant diffuses out of the reservoir through the membrane, thereby generating a concentration gradient in the collagen gel loaded in the sample well. The size, shape and position of the windows in the rectangular frames can influence the concentration profile in the sample well. In this work, each window had a diameter of 4 mm and was placed in the centre of the frame. In Figure 1.A, a 3D rendering shows the chamber in an exploded view, where the single components are highlighted, while in the assembled view in Figure 1.B the membrane supporting frames are housed in the chamber, separating the sample well and chemoattractant reservoir. The 3D renderings in Figure 1.A and 1.B are in scale with the actual device; the external size of the chamber is 52×25 mm (a scale bar is also reported in the figure). Chamber operation is as follows: 30 minutes after starting time-lapse image acquisition, 50 µg/ml IL-8 solution is carefully added to the chemoattractant reservoir by a tiny silicon tube connected to a syringe (this injection requires less than 1 minute and does not perturb image acquisition). This allows quantifying cell migration both prior to chemokine addition (random conditions) and after establishing a concentration gradient.

### Preparation of collagen gel

Neutrophil granulocytes were incorporated into 3D type I collagen lattices consisting of non-pepsinized rat-tail collagen (final concentration: 2.2 mg/ml; BD Biosciences). The collagen gels were prepared under sterile conditions by mixing cells suspended in RPMI 10% FBS (5×10^5^ cells/ml) with 0.1 M NaOH (9% v/v), RPMI 10× (7% v/v) and collagen solution (84% v/v). All components were kept on ice during the preparation, except for the cell suspension that was added at the end. Once cells were added, the solution was placed in one of the compartments of the chamber, previously sterilized in autoclave. The chamber was incubated at 37°C and 5% CO_2_ for 20 min to induce collagen polymerization. The collagen morphology after polymerization can be observed using confocal microscopy by exploiting the reflection of collagen fibres as shown in Figure 1.C.

### Time-lapse microscopy workstation

Microscopy observations were carried out by using a video microscopy time-lapse workstation [37]–[39]. The microscope (Zeiss Axiovert 200, 10× objective) is equipped with a high sensitivity cooled CCD camera (Hamamatsu Orca AG) and motorized stage, focus and filter wheel for automated 6D time-lapse imaging. It is worth mentioning that the assay proposed here can be run at higher magnifications and in epifluorescence or confocal microscopy as well. The whole workstation is driven by a control macro operated in the Labview software environment. The microscope is also equipped with a home-made incubator capable of keeping the sample temperature at 37±0.1°C in a saturated moisture atmosphere with 5% CO_2_. During the experiments, images in the sample well of the chemotaxis chamber were acquired at approximately 2 mm from the membrane along the y direction and at the center along the x direction (see axes orientations in Figure 1.B). The collagen gel was periodically scanned by optically imaging 5 layers separated by a 20 µm distance along the focus (z) direction within the collagen gel. The overall collagen gel thickness in the sample well was around 5 mm and the lowest z layer of the image stack was chosen to be approximately 400 µm from the bottom glass to avoid possible wall effects (such as surface-induced local orientation of collagen fibres). The frequency of acquisition was set to 1 image per minute, which was high enough to allow accurate tracking of cell trajectories based on the average speed of neutrophils (a few microns per minute).

### Chemotactic Factor (CF) concentration profile measurements

**Figure 2:**
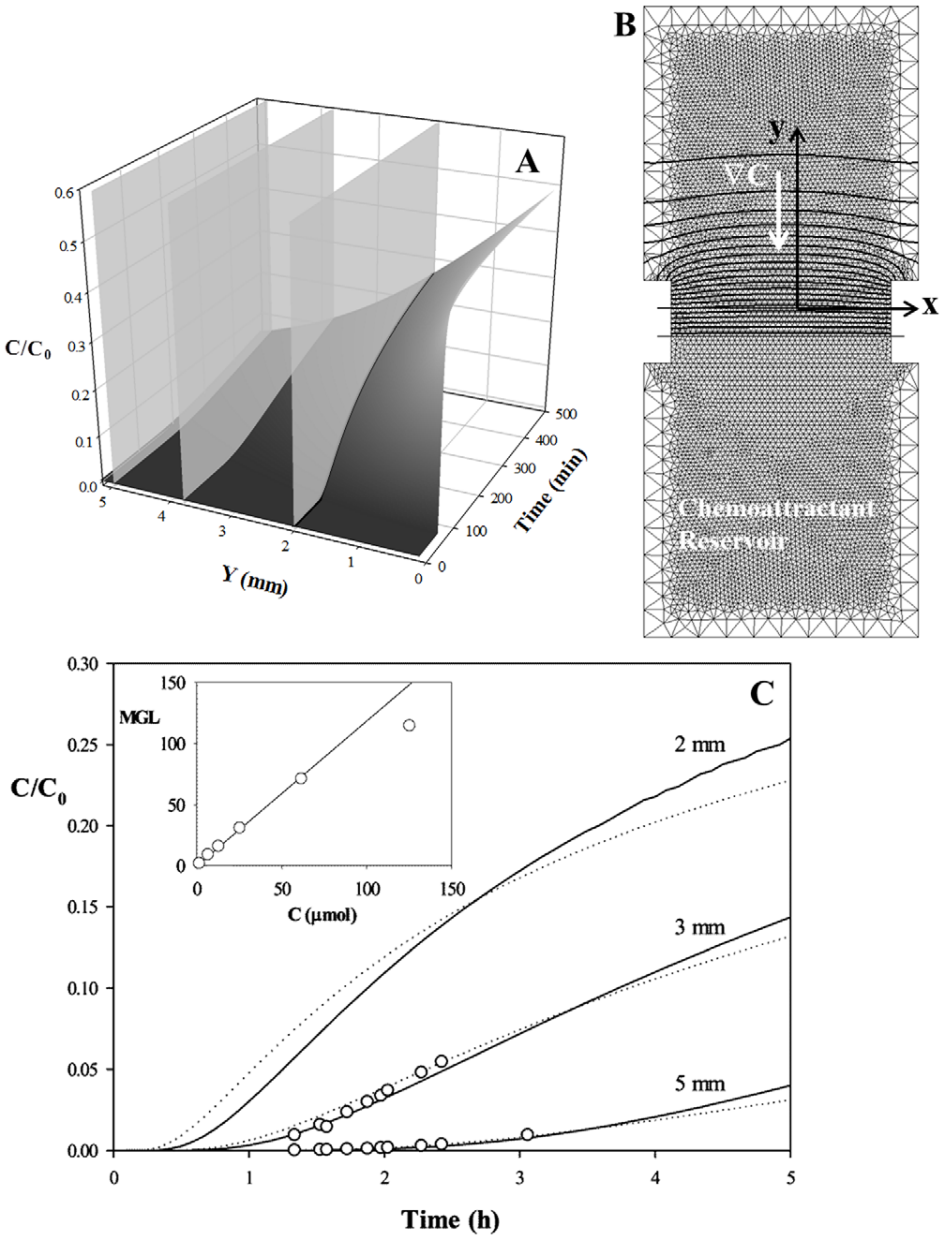
Chemoattractant diffusion. A: Evolution of the concentration profile as a function of space and time along the chemotaxis chamber according to Eq. 2. B: Finite element discretization of the chemotaxis chamber. Chemoattractant isoconcentration curves are superimposed to the discretization mesh. C: Chemoattractant concentration, normalized with respect to the initial concentration in the reservoir (C_0_) as a function of time at a distance of 2, 3 and 5 mm from the membrane. Dashed lines are the semi-infinite slab approximation (Eq. 2), continuous lines are the finite element simulations, circles are the experimental measurement of FITC-dextran concentration by mean grey intensity. Inset: Mean Gray Level measurement of epifluorescence images as a function of dextran concentration.

In order to quantify the chemoattractant concentration profile, a preliminary characterization of the chemotactic chamber was made by using fluorescently labelled dextran (FITC-dextran), having a molecular weight (10 kDa) comparable to that of IL-8 (8.5 kDa). The fluorescence intensity can be easily related to FITC-dextran concentration by means of a proper calibration, which was done by performing a set of experiments with FITC-dextran solutions having concentrations in the range 0.2 µM–120 µM. The solutions were loaded in a multiwell plate, which was incubated for 20 minutes to allow collagen gelification. Fluorescence images were acquired within each well by using the same optics, light power and CCD settings. The mean gray level, as calculated from the image histogram, was found to be a linear function of FITC-dextran concentration, as reported in the inset of Figure 2.C.

Based on the obtained calibration, FITC-dextran concentration profiles inside a collagen gel in the chemotaxis chamber were determined by epifluorescence time-lapse microscopy. The mean gray level of the image was measured as a function of time and position from the porous membrane, in order to characterize the propagation of the FITC-dextran front in the collagen gel. Images were acquired at a distance of 1 mm from each other, in the range from 2 to 5 mm from the membrane.

The concentration profile can be calculated from the classic Fickian diffusion model (Eq. 1) by considering the diffusion coefficient as a constant [40], [41] and the collagen matrix, where the FITC-dextran is diffusing, as a semi-infinite medium. According to the latter approximation it is assumed that the chamber wall at the opposite side of the membrane is at an infinite distance (hence, C|_y→∞_ = 0), while the concentration at the membrane is constant and equal to C_0_/2, where C_0_ is the initial concentration in the chemoattractant reservoir. The initial condition is that concentration is initially equal to 0 in the entire diffusion chamber [42]. By fitting the concentration profile (Eq. 2) obtained by solving Eq. 1 with the aforementioned boundary and initial conditions to the experimental data, the diffusion coefficient D can be calculated, and a value of 2·10^−6^ cm^2^/s is obtained.
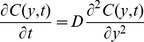
(1)

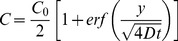
(2)


The expected concentration profile, normalized with respect to the concentration in the reservoir (C/C_0_), is reported in Figure 2.A as a function of space and time, according to Eq. 2.

Starting from this first estimate of the diffusion coefficient, a more detailed calculation was done by finite element analysis. The chemotactic cell was discretized in 2D using COMSOL multiphysics numerical simulation software (COMSOL Group). The chamber geometry was divided in 3 sections, according to the scheme reported in Figure 2.B, corresponding to the chemoattractant reservoir, membrane and collagen gel. The initial condition imposed in the calculation was the same as in the previous case, but the semi-infinite slab approximation was removed, and the effective size of the chamber was considered. Furthermore, no flow at the chamber walls was imposed. At the initial time stages the calculated concentration profiles were in good agreement with the ones obtained by assuming a semi-infinite medium. The concentration at the interface between the membrane and the collagen gel was approximately constant and equal to C_0_/2, as expected. In Figure 2.B contour lines of constant concentration are superimposed to the discretization mesh. The concentration profile turned out to be a function of the distance y from the membrane only, and substantially constant along the x direction. The influence of the size and position of the holes in the membrane supporting frames was minor along the x direction and just limited to a short distance from the membrane. The concentration at the wall opposite to the membrane eventually increases from the initial value of 0, and this is accompanied by a decrease of the concentration at the membrane-collagen interface. Therefore, at long times the semi-infinite medium approximation (Eq. 2) starts to fail. The time when Eq. (2) fails to describe the concentration profile calculated numerically depends on the length of the chamber. When considering the distance of the membrane from the opposite wall of the sample chamber, the longer the chamber, the wider the agreement. Since we are interested in investigating the motility of neutrophils, which are expected to have a fast response to the chemotactic stimulus, the chemotaxis experiments were performed for about 2 hours in a chamber having a length of 5 mm. The time evolution of the concentration at different distances from the membrane, corresponding to the predictions of Eq. (1) for y = 2, 3 and 5 mm, (highlighted by the gray plane slices in Figure 1.A), are compared in Figure 2.C (dashed lines) with numerical simulations (continuous lines) and the experimental measurements (circles). The time when the chemoattractant front reaches the sample increases with the distance from the membrane. In the time frame of interest to our work (i.e., 2 hours), the discrepancy between the approximate solution and the numerical simulation is minimal, and the former is in good agreement with experimental measurements. It is worth mentioning that the value of D obtained by fitting the numerical simulation with the experimental data (1.7·10^−6^ cm^2^/s, in the above defined time frame of interest) is quite close to the first estimate (2·10^−6^ cm^2^/s).

### Cell tracking analysis

Cell trajectories were reconstructed by means of a semi-automated image analysis macro [43], [44] based on standard software libraries (Image Pro Plus). The macro allows the user to identify each cell on the corresponding best focus layer at each time step. All the cells that were in focus in each layer were tracked. Cell position arrays were then processed by a Matlab script, in order to characterize quantitatively the effect of chemotaxis in terms of changes in motility parameters, i.e. the locomotory active fraction of a cell population, the average velocity of migration, and the cell orientation bias as quantified by the chemotactic index (I) [27]. The latter is used to quantitatively assess directional cell movement for observation times that are sufficiently long (greater than the cell persistence time), and is defined as the ratio between the net movement in the direction of the gradient and the total curvilinear length of the cell trajectory. It ranges from +1 (trajectory fully oriented towards the source of chemoattractant) to −1 (negative chemotaxis). I = 0 corresponds to a random motion where no preferential direction is observed.

The value of I at a given time is calculated by considering the motion of each cell from the beginning of the experiment (cumulative chemotactic index), and is determined by averaging the values for all the cells analysed, weighted on the curvilinear length of each cell trajectory.

### Statistical analysis

Data from about 70 cells were averaged in order to calculate motility parameters. In order to determine the percentage of cells moving randomly, we calculated the velocity modules and components of every cell, grouping the values in 5 minutes intervals, and running the t-test over each of the grouped distributions. The percentage of cells that failed the t-test, rejecting the null hypothesis (p<0.05), was considered to have a biased (non random) velocity distribution.

## Results

In order to validate our chemotaxis assay, several experiments were performed on human neutrophils freshly isolated from healthy donors. The proposed assay allows the observation and analysis of the locomotory behaviour of cells in the absence and presence of external pro-migratory chemotactic factors during the same experiment. Indeed, neutrophils were observed prior to and after the addition of 50 µg/ml IL-8 to the chemoattractant reservoir of the chamber (Figure 1). The samples were imaged every minute for 110 minutes and for each time point 70 cells were individually tracked by manually overlaying each cell contour. Here, we show representative results obtained from two different healthy donors (referred to as A and B), as an experimental validation of the proposed experimental technique.

### Qualitative analysis of cell motility: cell trajectory reconstruction

The response of neutrophils to IL-8 was first analysed qualitatively through the reconstruction of cell trajectories. Since collagen fibrils can become aligned (contact guidance) near the surface as the gel forms or within the gel as it compacts due to traction exerted by the entrapped cells, the possible contributions of these effects to directional cell migration and orientation need to be accounted for. Contact guidance is the phenomenon by which the extracellular matrix provides directional cues to the cells and directs the motility response via anisotropy in the microenvironment [45]–[47]. For instance, it has been shown that contact guidance from the alignment of collagen fibres promotes 3D migration of fibroblasts along the axis of collagen orientation [45], [46]. Recent studies [48] show that density and spatial alignments of (3D) collagen architecture, collagen concentration and the polymerization conditions, including pH and temperature, have considerable influence on scaffold structure and porosity and secondary impacts on cell morphology and behaviour including migration efficiency [49], [50]. We restricted our measurements to regions of the gel that are at least 400 µm away from the glass bottom and more than 1 mm from the top surface where it can be considered that any surface-induced alignment effects are negligible. Furthermore, we used low cell densities in order to prevent any significant gel compaction during the observation period.

**Figure 3:**
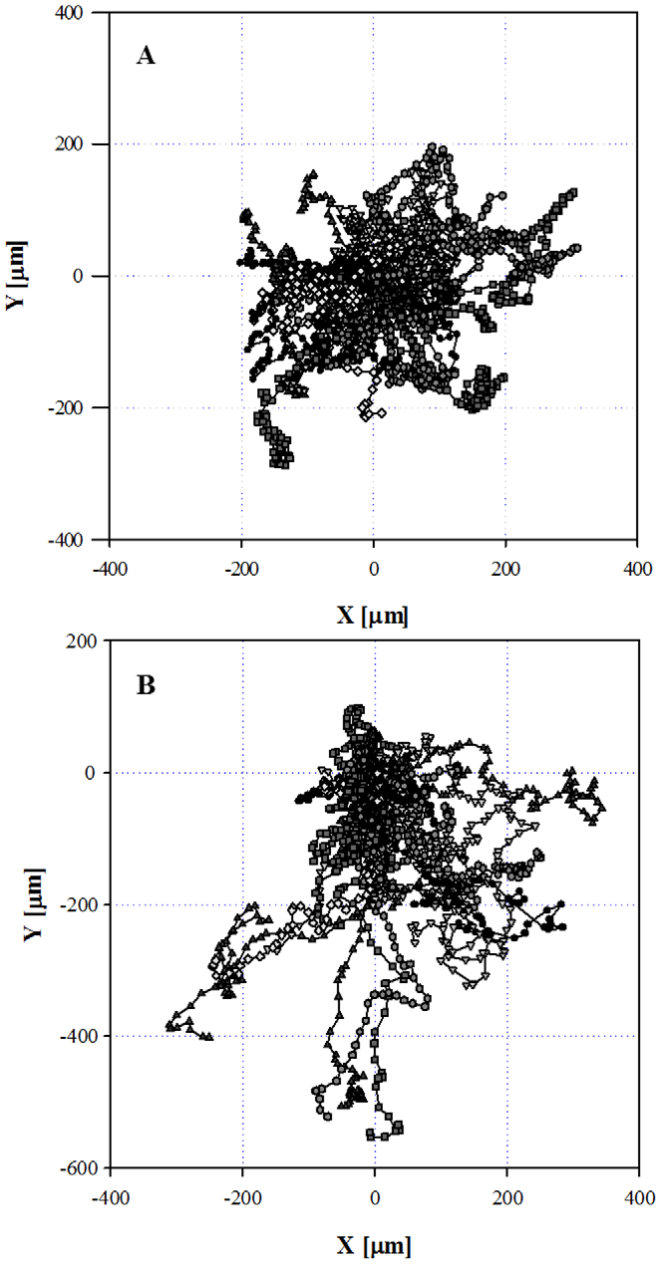
Cell trajectories projected on the XY plane and referred to the same origin. A: Random motion in absence of any chemical stimulus. B: In the presence of an IL 8 concentration gradient (C_0_ = 50 µg/ml) a preferential direction is qualitatively evident: the cells migrate towards the negative Y direction, i.e. in the direction of the membrane, towards the chemoattractant gradient.

Figure 3.A shows the trajectories of 54 cells, tracked for 110 minutes, labelled with symbols having different shapes and grey intensities, in isotropic conditions. Each trajectory is referred to the same initial position that coincides with the origin of the coordinate system. In this control experiment where no chemoattractant was added, cell trajectories were uniformly distributed in space (see Figure 3A), thus showing that cells were moving in a random orientation (i.e., no preferential direction of motion can be distinguished). Such random pattern confirms the absence of any matrix-mediated contact guidance effect. In the presence of the IL-8 concentration gradient, cell trajectories were instead directed towards the chemoattractant source as shown in Figure 3.B with results from 76 cells. Most of the cell paths have a preferential orientation toward the negative y axis, which is the direction of the concentration gradient.

**Figure 4:**
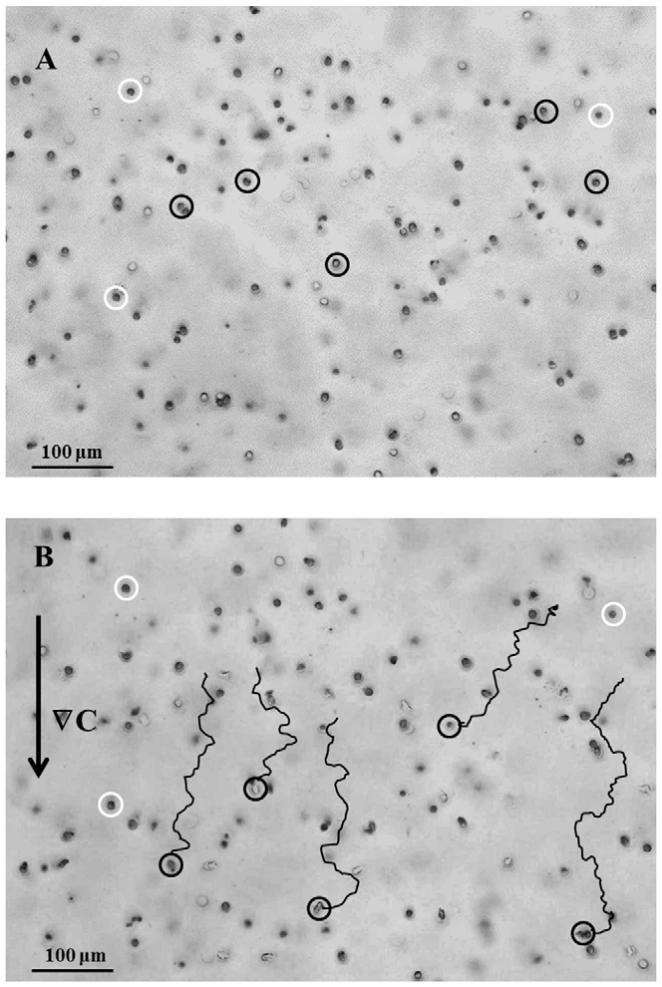
Images acquired at 5 consecutive focus positions within the collagen gel have been projected on the XY plane. The circles indicate the position of cells at two different times. Only the cells enclosed in a black circle move, while the white ones do not significantly change their position over the entire experiment. A: Time = 0. B: Time = 110 minutes. The complete trajectories described by motile cells are shown. The arrow indicates the direction of the chemoattractant concentration gradient ∇C.

Visual inspection of the time-lapse movie (provided as movie S1), shows that only a fraction of the cells did actually move during the entire experiment. In Figure 4 the z-projection of 5 consecutive images from a z-stack acquired within the cell seeded collagen gel is reported, showing the neutrophils at time 0 (Figure 4.A) and at time 110 minutes (Figure 4.B). The circles indicate the position of cells in focus at the two times, respectively. Only the cells enclosed in black circles exhibited a significant movement, while the white ones showed no appreciable change in their positions over the entire experiment. The complete trajectories described by the motile cells are drawn in Figure 4.B, where the direction of the chemoattractant concentration gradient ∇C is toward the bottom of the image as indicated by the arrow on the left. In the complete image sequence of the time-lapse experiment (see the movie S1) cells appear to move randomly in the first part of the experiment (t<30 min), while after the addition of the chemoattractant solution in the reservoir (t = 30 min) a preferential direction towards the chemoattractant source (i.e. towards the bottom side of the image) is observed.

### Quantitative analysis of cell motility: Evaluation of motile cell fraction, cell velocity and chemotaxis index

**Figure 5:**
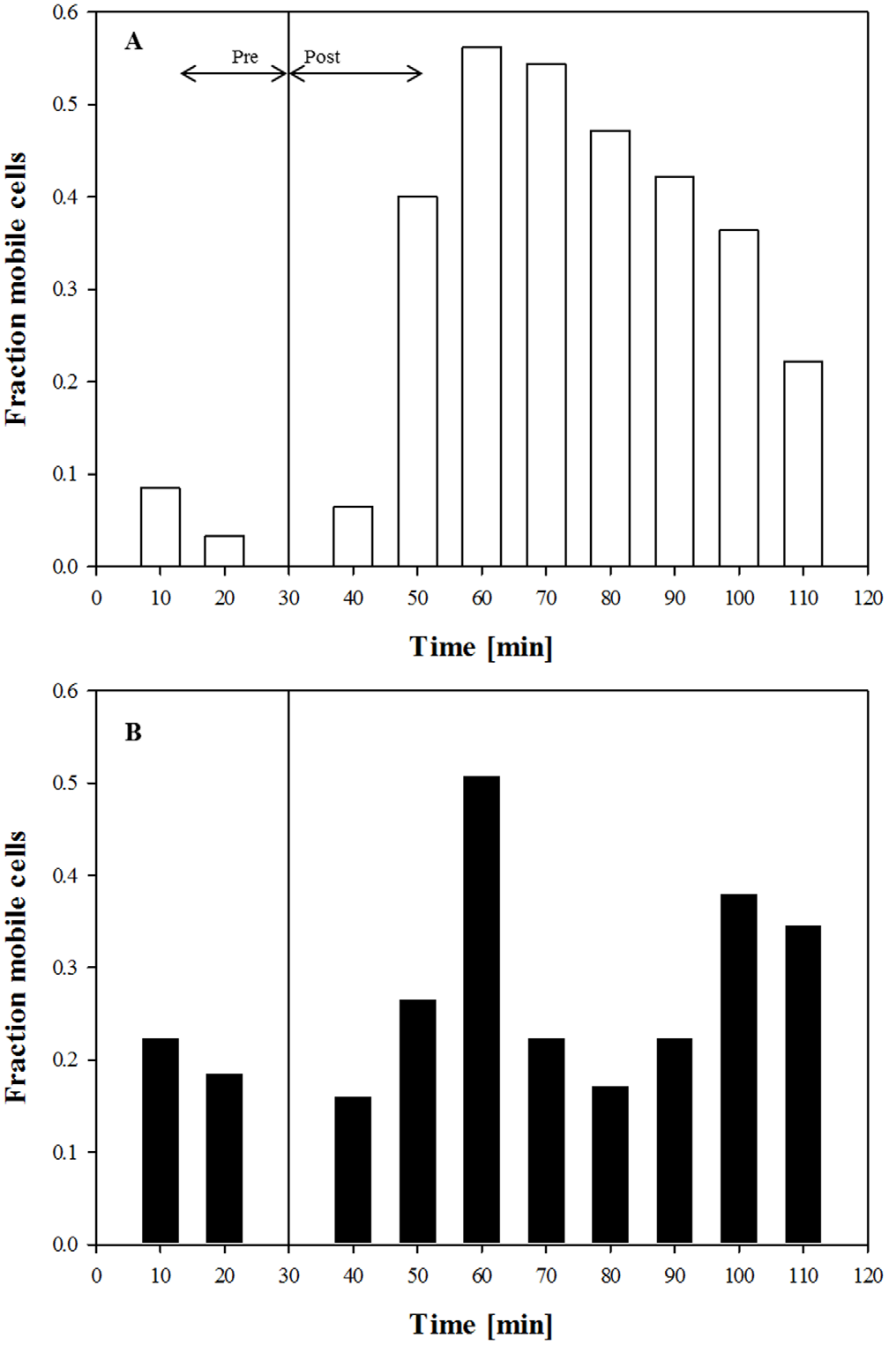
Fraction of motile cells as a function of the time for neutrophils A) from donor A, B) from donor B. The vertical line indicates the moment when the IL-8 solution was added to the chemoattractant reservoir.

A cell was considered motile in a given time interval if its total displacement exceeded its own diameter. This criterion was used to exclude values representing minor cellular displacements that might be caused by either the repositioning error of the microscope motorized stage or by external perturbations of the system. The fraction of mobile cells was calculated for a time interval of 10 minutes in presence and absence of IL-8 for donors A and B, and is reported in Figure 5 A and B, respectively; the vertical line at 30 minutes from the start of the experiment shows the time IL-8 solution was added. A different percentage of motile cells was clearly visible in unstimulated conditions (*Pre* IL-8) for the two samples with donor B showing a higher motility. However, in both donors the stimulation of the cells by IL-8 induced a significant increase in the number of motile cells as detected in the *Post* time stages. The evolution over time of the fraction of motile cells suggests that at short times cells are not affected by the presence of chemoattractant, but the motile fraction progressively increases, until a maximum is reached which is then followed by a decrease at later times when the influence of the chemoattractant starts to vanish.

**Figure 6:**
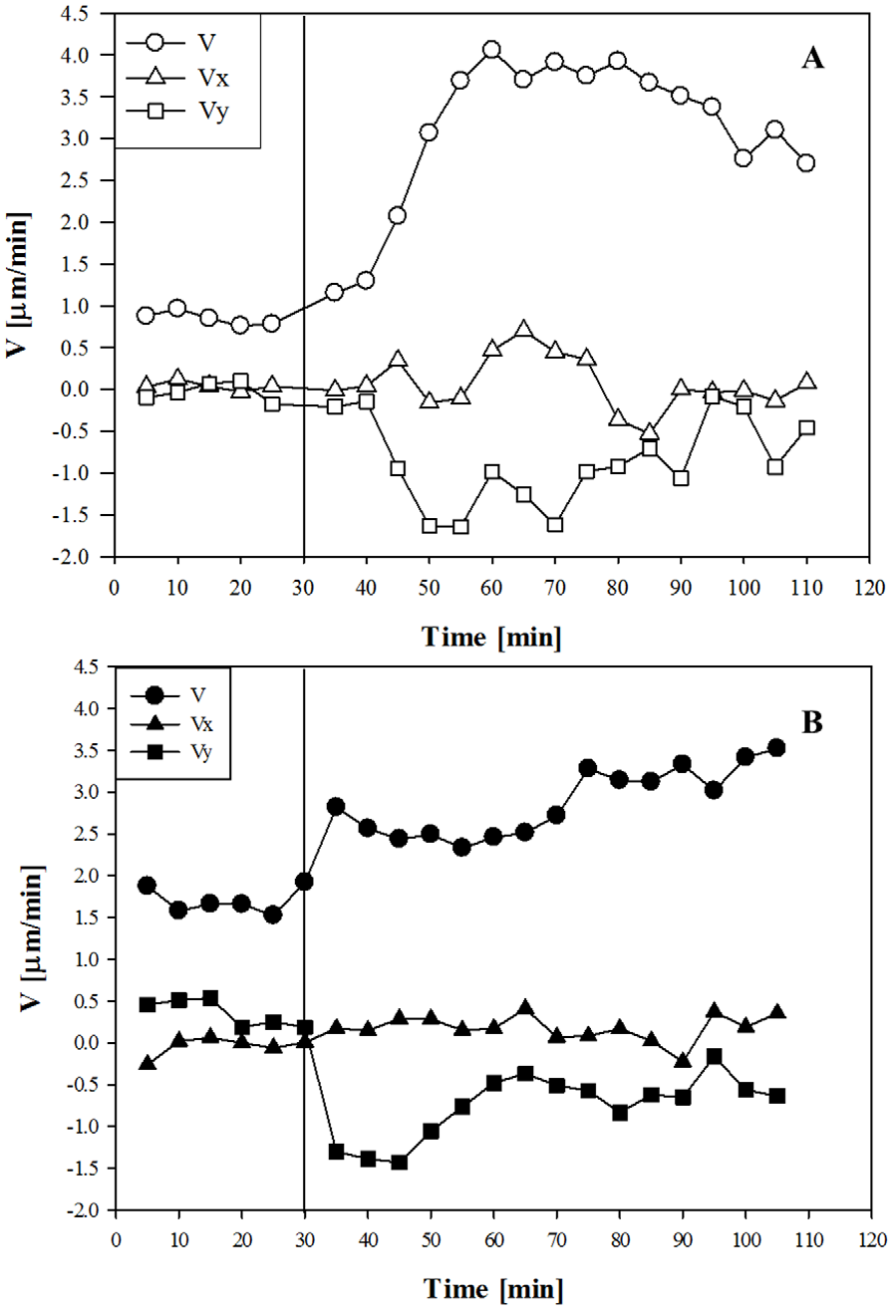
A: Average cell velocity components and modulus as a function of time for neutrophils from donor A. C: Average cell velocity components and modulus as a function of time for neutrophils from donor B. The vertical line indicates the moment when the IL-8 solution was added to the chemoattractant reservoir.

To further characterize the effect of chemokine gradient on neutrophils motility, the average velocity of cells was calculated for each time point and averaged along 5 minute intervals. In basal conditions, i.e. before adding the chemoattractant, cells from donor A displayed a lower average velocity than cells from donor B, i.e., 0.65 µm/min (Figure 6.A) *vs* 1.7 µm/min (Figure 6.B), respectively. This value increased after the addition of IL-8 in both donors, in agreement with the increment of the fraction of motile cells, thus confirming that the chemoattractant gradient elicits greater cell motility. Once again, the vertical line on the graphs in Figure 6 corresponds to the time point when the IL-8 solution was added. In Figure 6 the X and Y velocity components are also reported for both donors. The Y-component is the one showing the higher change after the IL-8 addition, evolving towards negative values, i.e. along the chemoattractant source, due to the orientation of the Y axis (see Figure 2.B). It is worth mentioning that for both donors the Y component of the average cell velocity showed a progressive decrease down to a negative peak, then slowly recovered towards basal values, a trend which confirms the presence of a transient peak in the chemotactic response. The statistical significance of these results was investigated by calculating the T-test for the velocity module and the X and Y velocity components. The percentage of cells that rejected the null hypothesis (i.e., zero average velocity) at the 5% significance level (p<0.05) is reported for both the donors in Figure S1. This parameter shows a transient peak for the Y component of the velocity after the addition of the IL-8 solution (as compared to the random conditions *Pre* IL-8), whereas the same peak is absent in the velocity module and the X component. This result provides further evidence that the effect of the IL-8 concentration gradient is to enhance cell movement mainly along the Y direction.

**Figure 7:**
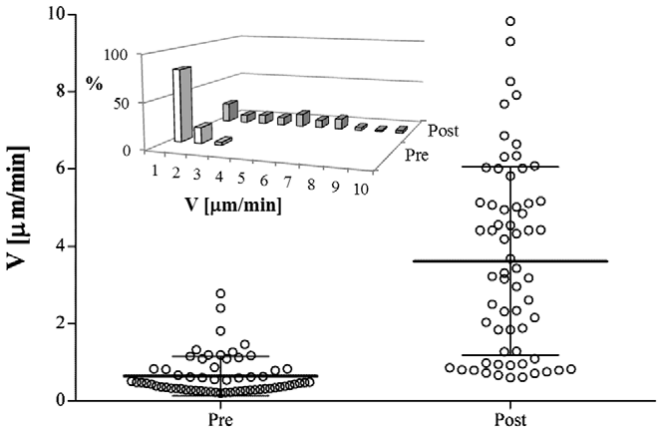
Average cell velocity module V calculated over the entire Pre IL-8 and Post IL-8 periods for each of the 63 cells from donor A. The continuous line is the average value, the error bar corresponds to the standard deviation for each population. Data are statistically significant (p<0.0001). In the inset the same data are plotted as a numerical distribution.

As a further statistical analysis we report in Figure 7 the average velocity module V calculated over the entire *Pre* IL-8 and *Post* IL-8 periods for each of the 63 cells from donor A. The continuous line refers to the average value, while the standard deviation is reported as the error bar. In the absence of IL-8 stimulation, i.e. for the initial time steps of the experiment (*Pre*), about 80% of the cells showed speeds in the range between 0 and 1 µm/min. The same calculation in the presence of the IL-8 concentration gradient (*Post*) showed that only about 20% of cells maintained a velocity ranging between 0 and 1 µm/min, while ∼60% of cells increased their speed in a range between 2 and 7 µm/min. The difference between the two populations is statistically significant (p<0.0001). In the inset of Figure 7 the velocity distribution calculated over the same data is also reported. A similar effect of IL-8 stimulation was observed in cells isolated from donor B (data not shown).

**Figure 8:**
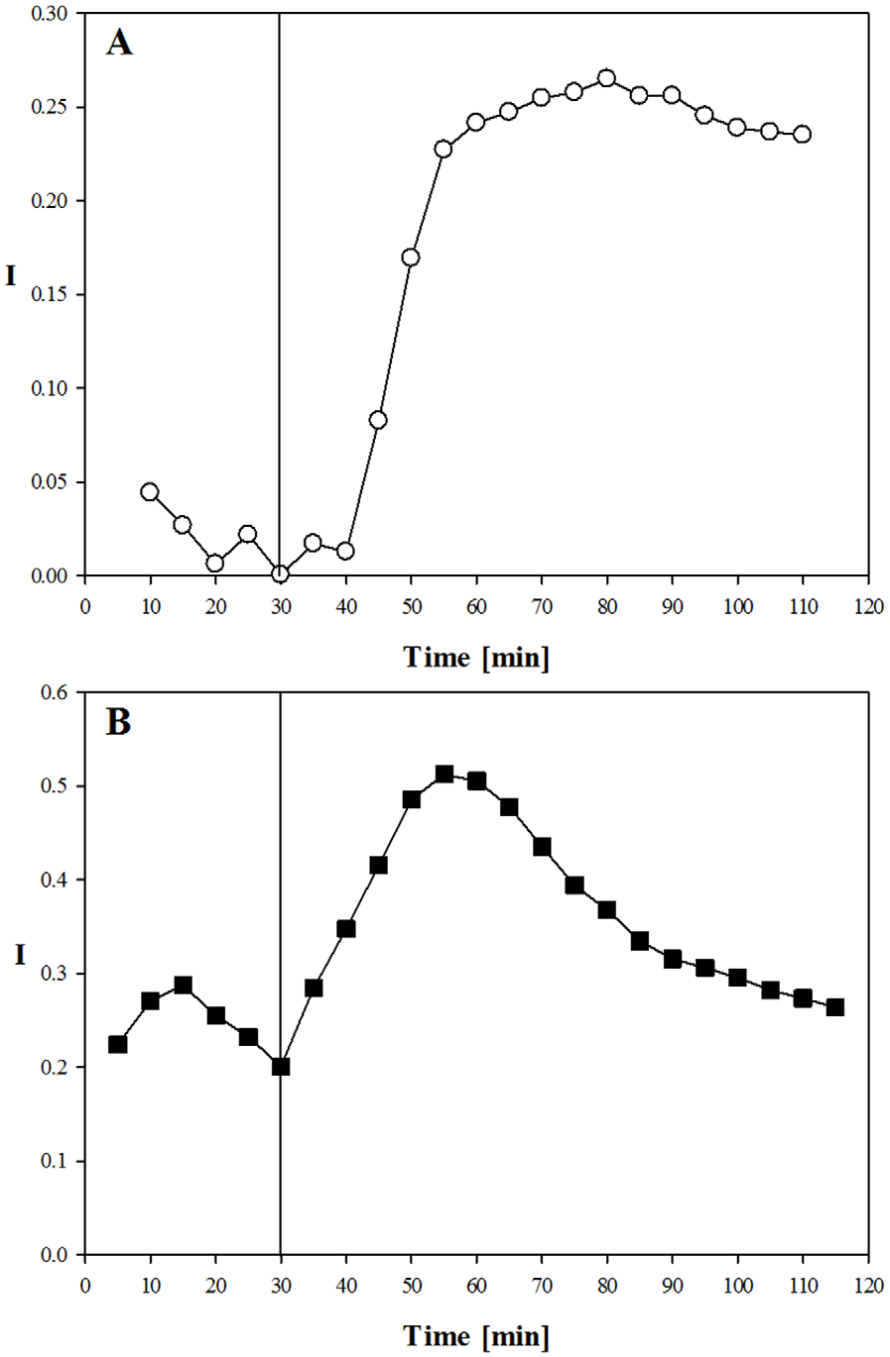
Chemotaxis index as a function of time for neutrophils A) from donor A, B) from donor B. The vertical line indicates the time when the IL-8 solution was added to the chemoattractant reservoir.

For each experiment, the cumulative chemotactic index (I) was calculated at regular steps of 5 minutes. Neutrophils from the two donors (Figure 8.A and 8.B) showed a different behaviour under basal conditions, fluctuating around 0 for donor A and around 0.25 for donor B. However, both samples showed a pronounced chemotactic response about 25 minutes after the addition of IL-8 (marked by the vertical line at time 30 minutes in the graphs). In particular, for donor A, I progressively increases reaching a value of 0.25 and then remains almost constant for the rest of the experiment. For donor B, I increases up to 0.5, then goes back to the basal value of 0.25 about an hour after the addition of IL-8. The chemotaxis index data are in agreement with the results obtained in terms of the fraction of motile cells and velocity.

## Discussion

The aim of this study is to develop an *in vitro* chemotaxis assay in tissue-equivalent collagen gels by using a direct-viewing chamber and a time-lapse microscopy and image analysis workstation. Our methodology allows the comparison of experimental results with control data, i.e. in the absence of chemotactic gradient, by analysing the motility of the same group of cells before and after chemoattractant addition.

Unlike other chemotaxis assays described in the literature, cell motility is investigated in dynamic conditions and in a physiologically relevant 3D environment consisting of collagen I, the predominant compositional and structural component of the extracellular matrix of connective tissues *in vivo*. Even though *in vitro* 3D collagen-based matrices lack the structural complexity and cross-links between molecules present in connective tissues [51], they reproduce the essential features of the structural characteristics of the network-like extracellular matrix and remain the most commonly used substrates to replace native collagen for *in vitro* cell migration studies [48], [52].

Human neutrophils, freshly isolated from peripheral blood of two volunteers, under the effect of IL-8, were used in this assay. To quantify the chemoattractant diffusion, a fluorescently labeled dextran (FITC-dextran) with a molecular weight comparable to that of the chemoattractant used in this work (IL-8) was used. The experimentally measured concentration profile was compared to the values calculated by numerical simulations, thus proving that the chemoattractant front can be approximated to a simple case of monodimensional diffusion in a semi-infinite slab and allowing us to determine the diffusion coefficient. Cell migration in a chemoattractant gradient was followed by time-lapse microscopy. Cell concentration was low enough to avoid possible contraction of the collagen matrix and distortion of the concentration profile. The response of neutrophils to IL-8 was first analysed qualitatively through reconstruction of cellular trajectory. In our chemotaxis chamber assay, the motion of cells in the XY plane in the absence of IL-8 showed a random orientation which was uniformly distributed in space, thus supporting the absence of any contact guidance effects. On the other hand, in the presence of IL-8 concentration gradients, cell trajectories were directed towards the chemoattractant source (Figure 3). Cell response was quantitatively characterized by calculating some key parameters of cell migration: chemotactic index, average cell velocity (both the module and the X, Y components), and fraction of motile cells. About 30 minutes after the addition of the chemoattractant to the reservoir, the fraction of motile cells (Figure 5) and the average cell velocity (Figure 6 and 7) showed an increase compared to the pre-treatment condition. Detailed analysis of cell velocity components (Figure 6) showed that the increase in velocity was mainly due to an increase in the y-component of the velocity, suggesting the presence of a net movement in the direction of the chemoattractant source, which is in agreement with the qualitative observation of the time lapse images and reconstruction of the cell trajectories (Figure 3 and 4, movie S1). The statistical significance of the velocity increase was also quantified by calculating the fraction of cells whose velocity rejected the null hypothesis (Figure S1). The x component of the velocity was not affected by the presence of the chemoattractant concentration gradient, and the movement along the x direction was substantially random. On the contrary, a peak along the y component of the velocity was measured, indicating that the movement along the direction of the chemoattractant concentration gradient was not random but directional. The presence of a chemotactic movement rather than a chemokinetic (i.e. random) effect was quantified by measuring the chemotactic index (Figure 8).

Neutrophils from two different donors displayed different behaviours both prior to and after the addition of IL-8. The differences in speed and fraction of motile cells might be related to different IL-8 circulating levels in the two donors. In fact, IL-8 production, which contributes to neutrophil activation and the development of acute inflammation [53], can be rapidly induced by several factors including bacterial and viral infections [54], [55]. Further experimental investigation over a wider donor population will allow a better understanding of this topic and will be a potential application of the proposed chemotaxis assay. However, for both donors a significant effect was observed in all the quantitative parameters investigated in the presence of an IL-8 concentration gradient, thus showing the efficacy of the experimental technique presented here. In particular, a transient increase in the fraction of motile cells, average velocity (specifically in the direction of the chemoattractant concentration gradient), and directionality, as measured by the chemotactic index, was found. A possible interpretation of the transient nature of the chemotactic response is the saturation of the cell membrane receptors [56], [57], which could be reached at a local chemoattractant concentration close to the dissociation constant of the cell receptors for IL-8 [58].

## Conclusions

We present an innovative methodology for the investigation of chemotaxis *in vitro* by time-lapse live cell imaging of cell movement under a controlled chemoattractant gradient in a direct viewing chamber. The chemotaxis chamber is autoclavable, reusable and is made of two compartments separated by a membrane which allows the diffusion of the chemoattractant from the reservoir to the 3D collagen matrix. The chemoattractant concentration profile can be experimentally quantified by fluorescence microscopy. The proposed technique allows comparison of chemotactic responses with control conditions, i.e. in the absence of any chemotactic gradient, during the same experiment and the analysis of the same group of cells under different conditions. Moreover, cell motility both in random conditions and under the presence of a chemotactic gradient can be investigated.

We validated our assay on polimorphonuclear lymphocytes in an IL-8 gradient. The reported results highlight a striking correlation between the presence of the chemokine and the increase in the percentage of motile cells, average cell velocity and chemotaxis index. Future applications of the proposed chemotaxis assay include a systematic comparison between the motility parameters of individual donors under the influence of different chemoattractants. Furthermore, the assay can be adapted to other cell types and may serve as a physiologically relevant method to study the directed migration of cells in a 3D environment in response to different chemoattractant agents.

## Supporting Information

Movie S1 **Directional migration of freshly isolated human neutrophils in 3D collagen matrix towards Il-8.** Images were taken at 10× magnification, and correspond to the Z projection of 5 layers reported at a reduced time and spatial resolution with about one frame every two minutes, in order to minimize the file dimension. The first 30 minutes, where each frame has a white overlay showing the progressive time and a dimension bar, are representative of the unstimulated condition where a limited fraction of cells move randomly. The IL-8 solution was added to the reservoir at t = 30 min. In the second part of the movie where the overlay is in black, cells are under the effect of an IL-8 concentration gradient. An increase in cell motility, that has been quantified in terms of average velocity and fraction of motile cells, and a preferential movement toward the source of the chemoattractant, i.e. towards the bottom part of the image, can be clearly noticed.
(AVI)

Figure S1 **T-test on velocity module and components.** Percentage of cells that rejected the null hypothesis at the 5% significance level (p<0.05), calculated for the velocity modulus and components, is reported. Y is the direction of the chemoattractant gradient. A and B panels are relative to data from donors A and B.
(TIF)
