# Supplementary figures and images for: A Novel Chemotaxis Assay in 3-D Collagen Gels by Time-Lapse Microscopy
Source: PLoS One. 2012 Dec 19;7(12):e52251. doi: 10.1371/journal.pone.0052251 (PMC3526591; doi:10.1371/journal.pone.0052251)

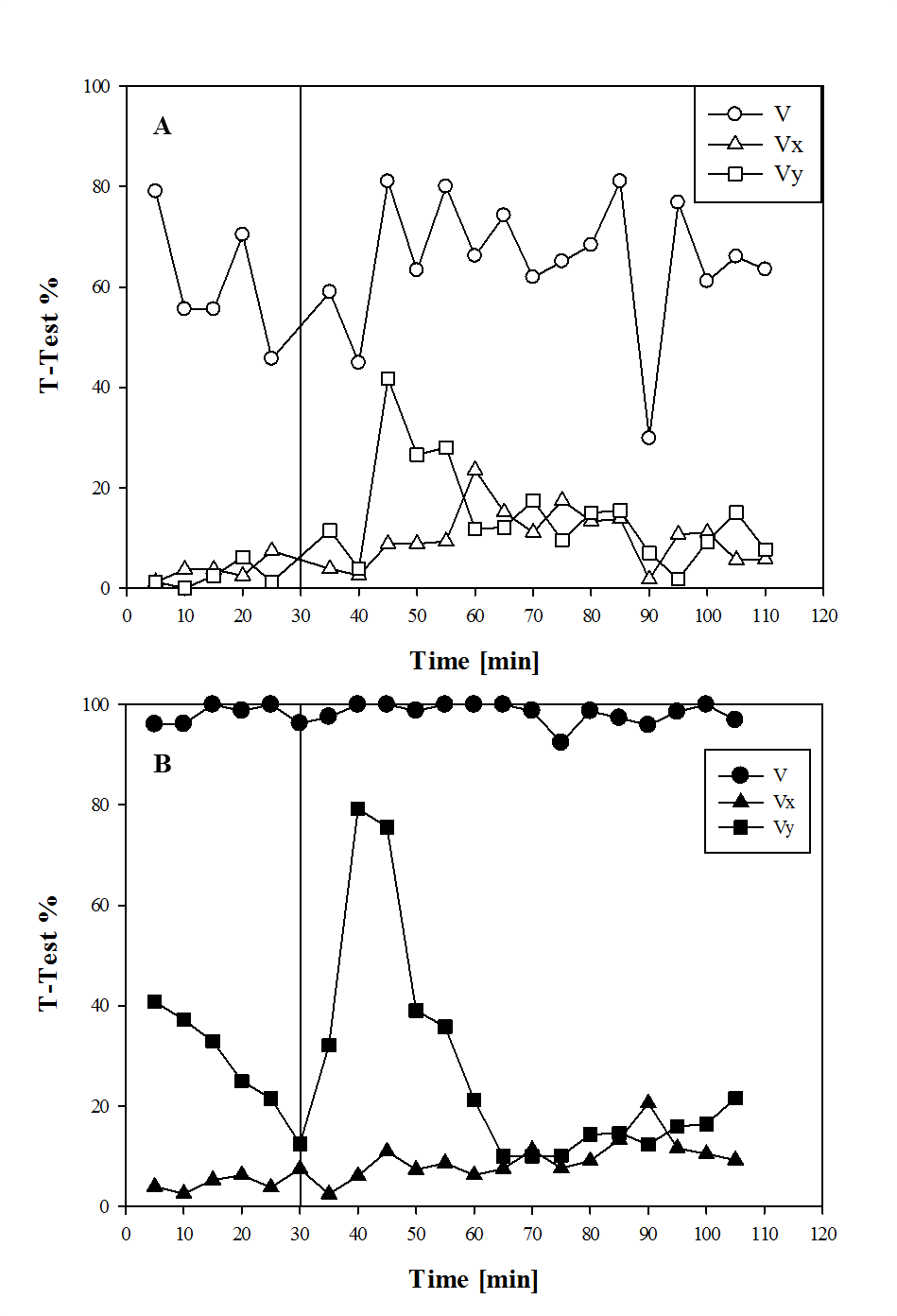

Supplement: Figure S1 — T-test on velocity module and components. Percentage of cells that rejected the null hypothesis at the 5% significance level (p<0.05), calculated for the velocity modulus and components, is reported. Y is the direction of the chemoattractant gradient. A and B panels are relative to data from donors A and B. (TIF) [file pone.0052251.s002.tif]
